# Attuali applicazioni della determinazione dei livelli plasmatici di copeptina in contesti non-endocrinologici

**DOI:** 10.1007/s40619-022-01180-8

**Published:** 2022-11-08

**Authors:** Alessandro Maria Berton, Emanuele Varaldo, Nunzia Prencipe, Francesca Rumbolo, Fabio Settanni, Andrea Benso, Silvia Grottoli

**Affiliations:** 1 Dipartimento di Scienze Mediche, Università degli Studi di Torino, Torino, Italia; 2 Divisione di Endocrinologia, Diabetologia e Metabolismo, A.O.U. Città della Salute e della Scienza di Torino, Torino, Italia; 3 Divisione di Biochimica Clinica, A.O.U. Città della Salute e della Scienza di Torino, Torino, Italia

**Keywords:** Arginin-vasopressina, Sindrome coronarica acuta, Scompenso cardiaco, Sepsi, Polmonite, Malattia del rene policistico autosomica dominante

## Abstract

Copeptina (CT-proAVP) rappresenta l’affidabile marker di secrezione dell’arginin-vasopressina (AVP) e nella pratica clinica endocrinologica viene impiegata per la diagnosi differenziale della sindrome poliurica-polidipsica. Negli ultimi anni, tuttavia, sono state indagate applicazioni alternative di tale glicopeptide in setting differenti. In questa rassegna sono esaminate, in particolare, le sue più interessanti proposte di utilizzo in ambito cardiovascolare, infettivologico e nefrologico.

**Informazioni Supplementari:**

La versione online contiene materiale supplementare disponibile su 10.1007/s40619-022-01180-8.

## Introduzione

Copeptina (CT-proAVP) è un peptide glicosilato, di 39 amminoacidi e approssimativamente 5 KDa di peso molecolare, che rappresenta la porzione C-terminale del polipeptide precursore dell’arginin-vasopressina (pre-proAVP) [1] e le cui funzioni biologiche sono per la maggior parte ancora sconosciute. In passato era stato proposto quale fattore di rilascio per la prolattina, ipotesi poi abbandonata, mentre è invece probabile una sua funzione quale molecola *chaperone-like* coinvolta nel corretto ripiegamento del pro-AVP.

Copeptina non presenta un ritmo circadiano significativo ma viene secreta dalla neuroipofisi, in quantità equimolari rispetto all’AVP, in risposta ai medesimi stimoli fisiologici. A tal proposito, molteplici trigger sono in grado di determinare il loro rilascio e taluni di essi risultano aspecifici (es. stress, dolore, nausea). Tuttavia, due sono i principali sistemi dedicati a regolare tale neurosecrezione ipotalamica: da un lato gli osmocettori centrali (presenti a livello dell’eminenza mediana) e periferici (localizzati nel tratto prossimale del tubo digerente) preposti a riconoscere sottili variazioni di osmolalità del fluido extracellulare (*extracellular fluid*, ECF); dall’altro i barocettori e volocettori dei vasi a grossa portata, rispettivamente ad alta pressione e di capacitanza, pronti a leggere eventuali alterazioni significative del volume circolante efficace (*effective circulating volume*, ECV) [2].

Una volta in circolo, copeptina vanta un’emivita quasi doppia rispetto all’AVP (pari a circa 40’), risultando almeno in parte soggetta a *clearance* renale a causa del suo ridotto peso molecolare. I valori mediani del glicopeptide nei soggetti sani e in condizioni di normale osmolalità plasmatica sono pari a 4,2 pmol/L (range 1–13,8 pmol/L), con una minima differenza tra i due sessi [1]. Dal punto di vista laboratoristico, copeptina è caratterizzata da una bassa variabilità preanalitica, vantando livelli stabili fino a 24 h nel campione ematico conservato a temperatura ambiente, così come nei campioni di plasma stoccato anche dopo molteplici cicli di scongelamento. Infine, il saggio in immunofluorescenza automatizzato su piattaforma KRYPTOR^®^ (Thermo Fisher Scientific, Hennigsdorf, Germany) rende necessarie minime quantità di plasma per l’analisi, garantendo inoltre rapidi tempi di analisi (circa 30’).

**Figure 1:**
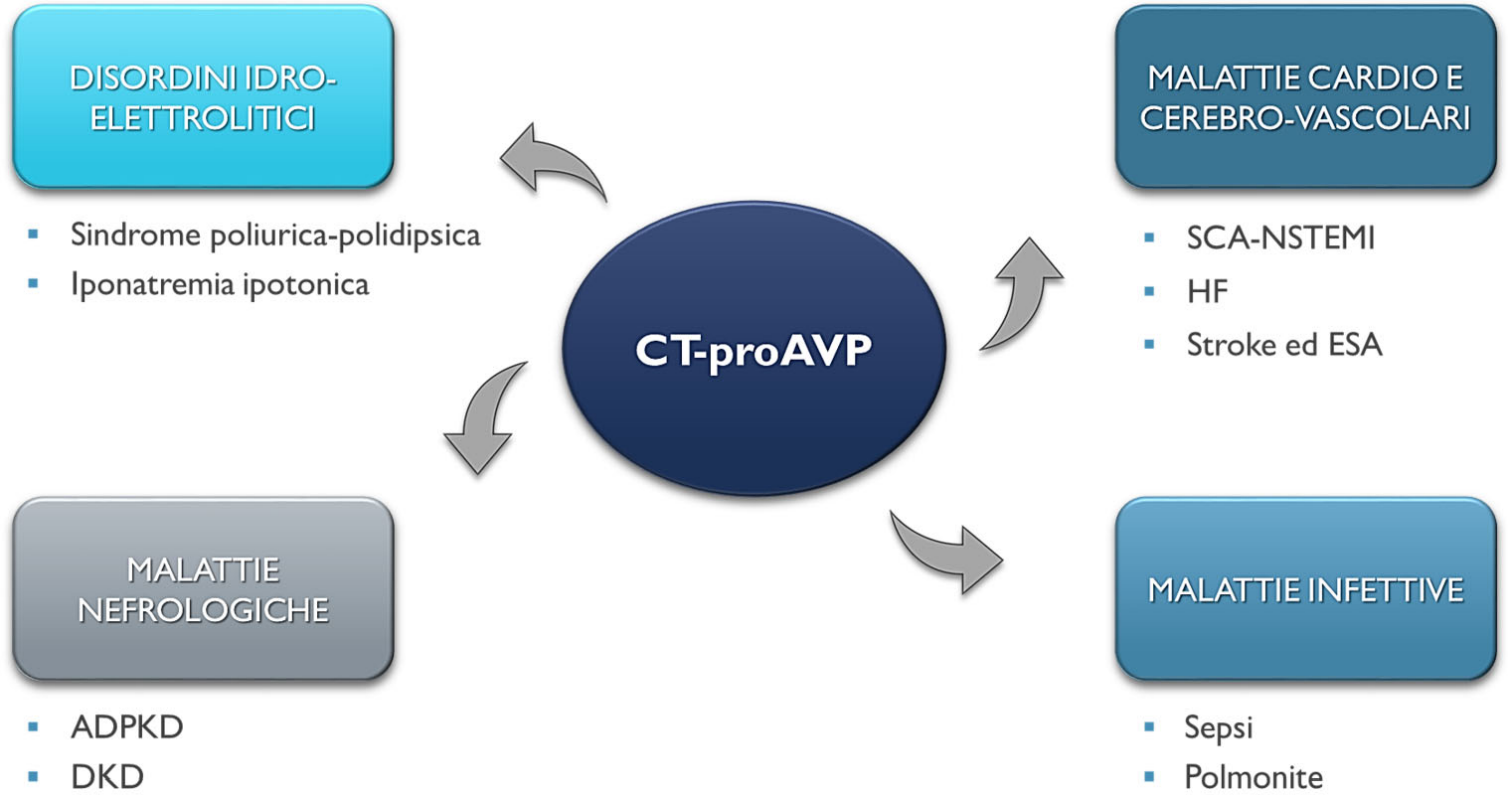

In ambito endocrinologico, copeptina ha un ruolo ben definito nella diagnosi differenziale della sindrome poliurica-polidipsica (Fig. 1) laddove livelli basali >21,4 pmol/L identificano con sicurezza i casi di diabete insipido nefrogenico e valori osmoticamente stimolati ≤4,9 pmol/L hanno un’ottima accuratezza nel diagnosticare le forme di diabete insipido centrale [1]. Inoltre, diversi studi hanno dimostrato la sua utilità nell’escludere i casi di iponatremia ipotonica secondari a polidipsia primaria, così come nell’identificare le forme francamente ipovolemiche di tale disturbo, o ancora nel distinguere quelle disvolemiche dalle euvolemiche. Tuttavia, in quest’ultimo ambito i cut-off disponibili in letteratura non risultano altrettanto affidabili e validati.

## Applicazioni in ambito cardio e cerebro-vascolare

Uno dei primi e più studiati ambiti di applicazione di copeptina al di fuori del *setting* endocrinologico è stato la rapida esclusione dell’infarto acuto del miocardio (IMA) in caso di dolore toracico sospetto per sindrome coronarica acuta (SCA) (Fig. 1). L’incremento immediato, seppur aspecifico, dei livelli di copeptina all’insorgenza della sintomatologia cardiaca risulta infatti più precoce rispetto a quello dei tradizionali biomarcatori di danno miocardico (*creatine kinase-MB*, CK-MB; *cardiac troponin*, cTn); ciò nonostante è bene ricordare come tale aumento non risulti significativo in caso di angina instabile [3]. Dal punto di vista fisiopatologico, il presupposto del marcato aumento dei livelli del glicopeptide contestualmente a un evento ischemico risiede nella pronta risposta ipotalamica allo stress, a cui l’AVP prende parte in modo sinergico al CRH (*corticotropin releasing hormone*) (Tabella 1) [2]. Una *dual-marker strategy* (DMS), che comprendesse la determinazione di copeptina in associazione a quella di uno dei biomarcatori cardiaci tradizionali, si è dimostrata in più occasioni in grado di aumentare significativamente la loro accuratezza riducendo, inoltre, i tempi necessari per l’esclusione di un *non-ST-elevation myocardial infarction* (NSTEMI) (Tabella 2) [3]. Le linee guida della European Society of Cardiology (ESC) raccomandano, pertanto, l’utilizzo della DMS attraverso determinazione dei livelli di copeptina qualora non fossero disponibili saggi ultrasensibili per la determinazione della troponina cardiaca (*high-sensitivity cardiac troponin*, hs-cTn) [4]. Infatti, l’accuratezza praticamente sovrapponibile della hs-cTn rispetto alla DMS ha condotto sinora a un suo scarso utilizzo nella pratica clinica dei Centri dove fossero disponibili metodiche di ultima generazione; ciò nonostante, la sua implementazione potrebbe rappresentare un valore aggiunto anche in queste situazioni [5]. Recentemente, inoltre, l’utilizzo di *score* compositi comprendenti copeptina ha suscitato ulteriore interesse in merito alla diagnosi differenziale tra IMA di tipo 1 (secondario a trombosi da rottura di placca ateromasica) e di tipo 2 (associato a discrepanza tra offerta e domanda di ossigeno da parte del miocardio nel contesto di una condizione patologica acuta) [6].

**Table 1:** 

	V1aR (V1R)	V2R	V1bR (V3R)
Localizzazione	Sistema cardiovascolare, SNC, apparato iuxtaglomerulare, tubuli renali, piastrine	Porzione spessa del tratto ascendente dell’ansa di Henle, tubulo convoluto distale, dotto collettore; endotelio vascolare	Anteroipofisi, SNC, isole pancreatiche
*Signaling*	*Gq protein*, PLC-IP3	*Gs protein*, adenilato ciclasi-PKA	*Gq protein*, PLC-IP3
Effetti principali	Vasocostrizione splancnica, glicogenolisi, aggregazione piastrinica, controllo pressione arteriosa e temperatura corporea	Fosforilazione e trascrizione AQP2; stimolazione Na^+^/K^+^-ATPasi, NKCC2 ed ENaC; fosforilazione UT-A1 e A3	Stimolazione rilascio ipofisario di ACTH

**Table 2:** 

	Valori di copeptina	Interpretazione
SCA-NSTEMI	>10 pmol/L [3]	Cut off per rapida esclusione dell’IMA in associazione a cTn in occasione di dolore toracico
HF	22 pmol/l [7]	Livelli mediani riportati negli studi condotti su pazienti con HF
Sepsi	25,3–79,5 pmol/L [12]	Valori mediani riportati negli studi condotti su pazienti con sepsi
CAP	>29 pmol/L [14]	Cut-off ottimale per predire la mortalità a 28 giorni
ADPKD	2,9 pmol/L [20]	Livelli mediani di copeptina in un ampio studio condotto su pazienti con ADPKD ed eGFR conservato
DKD	12,7–13,6 [23]	Livelli basali mediani di copeptina nel terzo terzile, associati in pazienti con DM tipo 2 a raddoppio dei livelli di creatinina e progressione a ESRD a 6 anni

Sempre in ambito cardiologico, ma al di fuori del regime di urgenza, un’altra possibile applicazione di copeptina è rappresentata dallo scompenso cardiaco (*heart failure*, HF) (Fig. 1). In questo caso, il meccanismo fisiopatologico alla base della persistente secrezione di AVP, così come in altre condizioni patologiche ipervolemiche, è da attribuire in buona parte alla percezione da parte dei barocettori dei grossi vasi di un ridotto ECV, a causa di una cronica riduzione della gittata cardiaca. L’effetto rimodellante e profibrotico sul miocardio, esercitato da livelli di AVP e angiotensina II cronicamente elevati attraverso i propri recettori V1aR e AT1 (Tabella 1), comporta inoltre evidenti effetti avversi a lungo termine con aumentata morbilità e mortalità in questi pazienti.

In tale contesto infatti, copeptina rappresenta un marcatore prognostico indipendente di mortalità [7], sia per insufficienza cardiaca (HR 1,76; IC 95% 1,33–2,33) che per tutte le cause (HR 1,03; IC 95% 1,01–1,05) [8]. I livelli circolanti del glicopeptide (Tabella 2) predicono inoltre la necessità di ricovero per riacutizzazione di HF, nonché quella di trapianto cardiaco o di impianto di *left ventricular assist device* (LVAD) nelle forme avanzate di malattia (HR 3,28; IC 95% 1,66–6,50) [9]. In questi pazienti copeptina fornisce dunque informazioni prognostiche simili, se non superiori, a quelle offerte dalla determinazione di NT-proBNP, in particolare quando combinata ai livelli di quest’ultimo, a quelli di hs-cTn o di natremia.

Infine, copeptina è stata estesamente studiata nel contesto dello *stroke* e dell’emorragia subaracnoidea (ESA) (Fig. 1). In particolare, livelli elevati del biomarcatore sono stati osservati sia a seguito di *stroke* ischemico che di emorragia intra-parenchimale in modo, peraltro, non distinguibile dall’ESA, e correlavano con la mortalità dei pazienti [10]. Pertanto, in maniera indipendente dal tipo di danno cerebrovascolare intercorso, il valore di copeptina in tale contesto risulta al momento prognostico piuttosto che diagnostico. In particolare, i livelli del glicopeptide misurati entro 24 ore da uno *stroke* ischemico collaborano a predire in maniera più accurata disabilità e mortalità a tre mesi dall’evento (*CoRisk score*) [11].

## Applicazioni nelle malattie infettive

Anche in occasione di stati infiammatori acuti l’AVP viene secreta a livello ipotalamico, supportando il CRH nello stimolo dell’asse *hypothalamic-pituitary adrenal axis* (HPA) e partecipando alla regolazione dei processi flogistici (Tabella 1) [2]. È logico, pertanto, aspettarsi valori aumentati di copeptina in corso di stati infettivi sistemici, come la sepsi, o di infezioni delle basse vie respiratorie (Fig. 1). In tale contesto è stato diffusamente dimostrato come i livelli circolanti di copeptina correlino con le condizioni cliniche del paziente e la sua prognosi, predicendone talora la risposta ai trattamenti [12–14].

È infatti assodato come nei pazienti ricoverati in area critica i valori di copeptina aumentino progressivamente da un minimo osservabile in coloro affetti da *systemic inflammatory response syndrome* (SIRS) in assenza di infezione, verso livelli chiaramente superiori in caso di sepsi o *shock* settico [12]; contestualmente, livelli del biomarcatore più elevati all’ingresso in Unità di Terapia Intensiva (UTI) si associano a un’aumentata mortalità sia intra- che extra-ospedaliera [13]. Sebbene, quindi, il valore assoluto di copeptina possa di per sé aiutare i clinici nell’identificare i casi più a rischio di *outcome* negativo, è auspicabile una sua progressiva integrazione ai comuni *score* prognostici attualmente in uso in UTI. In particolare, nonostante i progressi fatti nella gestione della terapia antibiotica e delle problematiche emodinamiche ad essa associate, la sepsi rappresenta ancora una delle principali cause di mortalità in area critica. Questa condizione viene generalmente diagnosticata all’accesso tramite *score* quali il *sequential organ failure assessment* (SOFA) o il quick-SOFA (qSOFA), che valutano l’entità del danno d’organo e le condizioni cliniche dei pazienti. Tali *score*, tuttavia, oltre a risultare spesso indaginosi e di non rapida applicazione nella pratica clinica, non permettono da soli di ottenere sempre un’affidabile categorizzazione prognostica.

Parimenti, anche gli *score* attualmente indicati per identificare i pazienti affetti da *community-acquired pneumonia* (CAP) meritevoli di ricovero ospedaliero, quali ad esempio il *pneumonia severity index* (PSI) e il CURB-65, non sono sempre caratterizzati da un elevato potere discriminatorio nel distinguere le forme più severe di malattia. Anche in quest’ultimo ambito, i livelli plasmatici di copeptina valutati al momento del ricovero dimostrano di correlare indipendentemente con la prognosi di questi pazienti, almeno eguagliando le *performance* di tali *score* [14, 15].

Recentemente, infine, il ruolo prognostico di copeptina è stato valutato anche in pazienti con polmonite da Sars-CoV2 (COVID-19). Nonostante i livelli del glicopeptide non permettessero di distinguere questi casi da infezioni delle basse vie respiratorie secondarie ad altra eziologia [16], anche in tale contesto copeptina si è dimostrata un accurato predittore di complicanze intra-ospedaliere e mortalità [16, 17].

## Applicazioni in ambito nefrologico

La trattazione delle possibili applicazioni di copeptina in ambito nefrologico richiede la preliminare assunzione dell’impatto determinante sui suoi livelli circolanti di un’eventuale riduzione del filtrato glomerulare stimato (*estimated glomerular filtration rate*, eGFR), in quanto specchio della sua *clearance* renale. I dati attualmente disponibili sono, infatti, complessivamente a favore dell’attendibilità della valutazione di copeptina quale marker di secrezione dell’AVP fino a riduzioni di eGFR di grado moderato-severo (indicativamente eGFR >30 ml/min/1,73 mq), mentre cautela deve essere applicata in caso di concomitante *chronic kidney disease* (CKD) di grado 4 e 5 [18].

L’*autosomal dominant polycystic kidney disease* (ADPKD) rappresenta il principale ambito di applicazione di copeptina in *setting* nefrologico (Fig. 1). In questa malattia, secondaria a mutazioni dei geni PKD1 o PKD2, il razionale fisiopatologico alla base del monitoraggio dei livelli di copeptina è infatti particolarmente forte. La produzione di adenosina monofosfato ciclico (AMPc), un potente stimolatore della formazione e della crescita delle caratteristiche cisti, è direttamente favorita dall’interazione dell’AVP con il suo recettore V2R, di cui rappresenta un mediatore intracellulare (Tabella 1). I livelli circolanti di AVP potrebbero, peraltro, risultare cronicamente elevati in questi pazienti, in maniera proporzionale allo stato di avanzamento della malattia, a causa del sovvertimento dell’architettura midollare renale, con conseguente ridotta capacità di riassorbimento di acqua libera da soluti dalle preurine. Purtuttavia, ad oggi non vi sono evidenze che copeptina possa rappresentare un *biomarker* diagnostico di tale condizione, in quanto solo lievemente elevata in questi pazienti e più in generale non chiaramente associata a una specifica causa sottostante di CKD (Tabella 2) [19]. La caratterizzazione del glicopeptide come *marker* prognostico di rapida progressione (definita come aumento del *total kidney volume*, TKV, e riduzione dell’eGFR), sembra invece confermata da studi a lungo termine condotti su ampie popolazioni, anche tenendo conto di possibili fattori interferenti [20]. Infine, l’eventuale e auspicabile utilizzo di copeptina come predittore di risposta alla terapia medica con tolvaptan, antagonista recettoriale selettivo V2R in grado di ridurre la progressione di malattia nei pazienti affetti da ADPKD, richiede ad oggi ulteriori conferme [21].

Sempre in *setting* nefrologico, copeptina è stata studiata nel contesto della malattia renale diabetica (*diabetic kidney disease*, DKD) (Fig. 1). I livelli circolanti del glicopeptide sono lievemente più elevati nei pazienti affetti da diabete mellito (DM) di tipo 1 e di tipo 2 rispetto ai soggetti sani [22], anche se le basi fisiopatologiche di tale fenomeno non sono ancora completamente chiarite. A tal proposito, sono state ipotizzate tanto una relativa riduzione dell’ECF secondaria a glicosuria, quanto una possibile alterazione della sensibilità osmocettoriale ipotalamica in questi pazienti. Recenti evidenze, raccolte da studi osservazionali di grandi dimensioni condotti su pazienti con DM, hanno dimostrato una chiara associazione tra copeptina e valori di albuminuria [23], oltre a un forte valore prognostico del glicopeptide circa il rischio di sviluppare insufficienza renale terminale (*end-stage renal disease*, ESRD) a diversi anni di distanza (Tabella 2) [23, 24]. Più in generale, stanno emergendo evidenze a favore del ruolo prognostico di copeptina in merito al rischio di eventi cardiovascolari maggiori nei pazienti con DM, talora dimostrato anche indipendentemente dai valori di eGFR [24, 25].

## Conclusioni

Le applicazioni diagnostiche, in setting non endocrinologici, della determinazione plasmatica di copeptina sono ad oggi sostanzialmente rappresentate dall’esclusione di un NSTEMI nel contesto della SCA. Sono invece necessarie, al momento, ulteriori evidenze per poterne immaginare un impiego, sempre a scopo diagnostico, in ambito infettivo o nefrologico.

Dal punto di vista prognostico, copeptina ha invece dimostrato la potenzialità di un efficace impiego in una varietà di ambiti clinici differenti. Tra questi, nella pratica clinica cardiologica, meritano particolare attenzione la predizione di riacutizzazioni di scompenso cardiaco cronico o di necessità di trattamenti avanzati per i casi non più responsivi a terapia medica. In ambito infettivologico la valutazione di copeptina potrebbe in futuro integrare stabilmente alcuni degli *score* prognostici di comune utilizzo in area critica per la stratificazione di pazienti affetti da sepsi o polmonite.

Infine, nel setting nefrologico si prospettano ugualmente rilevanti opzioni di utilizzo, che necessitano, tuttavia, di ulteriori evidenze scientifiche sia in merito all’attendibilità del dosaggio in corso di CKD, sia sul reale significato patogenetico di valori elevati di AVP in questo ambito.

## Informazioni Supplementari

I link al materiale elettronico supplementare sono elencati qui sotto.

## Dichiarazioni etiche

### Conflitto di interesse

Alessandro Maria Berton ha ricevuto compensi da Thermo Fisher Scientific per passate collaborazioni editoriali. Gli altri autori, Emanuele Varaldo, Nunzia Prencipe, Francesca Rumbolo, Fabio Settanni, Andrea Benso e Silvia Grottoli dichiarano di non avere conflitti di interesse.

### Consenso informato

Lo studio presentato in questo articolo non ha richiesto sperimentazione umana.

### Studi sugli animali

Gli autori di questo articolo non hanno eseguito studi sugli animali.
