# Supplementary material for: Attuali applicazioni della determinazione dei livelli plasmatici di copeptina in contesti non-endocrinologici
Source: L'Endocrinologo. 2022 Nov 8;23(6):592–7. [Article in Italian] doi: 10.1007/s40619-022-01180-8 (PMC9641695; doi:10.1007/s40619-022-01180-8)
Supplement: Supplementary file 1 [file 40619_2022_1180_MOESM1_ESM.doc]

**Schede di autovalutazione**

**1. La determinazione dei livelli plasmatici di copeptina in caso di dolore toracico sospetto per sindrome coronarica acuta:**

a. si sostituisce alla determinazione dei tradizionali marcatori di necrosi miocardica

b. non è suggerita dalle linee guida della Società Europea di Cardiologia

c. in associazione alla determinazione di troponina cardiaca, rappresenta la dual marker strategy raccomandata per l’esclusione del NSTEMI in assenza di saggi ad alta sensibilità per la troponina stessa

**2. La determinazione dei livelli plasmatici di copeptina nell’ambito dello scompenso cardiaco:**

a. vanta un valore prognostico paragonabile a quello dell’NT-pro-BNP

b. vanta un valore diagnostico paragonabile a quello dell’NT-proBNP all’accesso in Pronto Soccorso nell’escludere una dispnea di origine cardiogena

c. permette di identificare i casi secondari a cardiopatia ischemica

**3. La determinazione di copeptina in ambito infettivologico:**

a. consente di distinguere accuratamente le polmoniti atipiche dalle altre infezioni delle basse vie respiratorie

b. ha dimostrato un forte potere prognostico dal momento dell’accesso dei pazienti in ospedale sia in caso di sepsi che di polmonite

c. non ha purtroppo un razionale fisiopatologico

**4. La valutazione dei livelli plasmatici di copeptina in pazienti affetti da insufficienza renale cronica:**

a. permette di distinguere la condizione patologica alla base della riduzione del filtrato glomerulare

b. deve tenere conto di un verosimile aumento dei livelli circolanti del glicopeptide, in caso di riduzione del filtrato glomerulare di grado severo, secondario a ridotta clearance renale

c. non possiede valore prognostico in merito alla progressione verso l’insufficienza renale terminale
