# Supplementary material for: Attuali applicazioni della determinazione dei livelli plasmatici di copeptina in contesti non-endocrinologici
Source: L'Endocrinologo. 2022 Nov 8;23(6):592–7. [Article in Italian] doi: 10.1007/s40619-022-01180-8 (PMC9641695; doi:10.1007/s40619-022-01180-8)
Supplement: Supplementary file 2 [file 40619_2022_1180_MOESM2_ESM.doc]

**RISPOSTE**

**1. La determinazione dei livelli plasmatici di copeptina in caso di dolore toracico sospetto per sindrome coronarica acuta:**

c. in associazione alla determinazione di troponina cardiaca, rappresenta la dual marker strategy raccomandata per l’esclusione del NSTEMI in assenza di saggi ad alta sensibilità per la troponina stessa

**2. La determinazione dei livelli plasmatici di copeptina nell’ambito dello scompenso cardiaco:**

a. vanta un valore prognostico paragonabile a quello dell’NT-pro-BNP

**3. La determinazione di copeptina in ambito infettivologico:**

b. ha dimostrato un forte potere prognostico dal momento dell’accesso dei pazienti in ospedale sia in caso di sepsi che di polmonite

**4. La valutazione dei livelli plasmatici di copeptina in pazienti affetti da insufficienza renale cronica:**

b. deve tenere conto di un verosimile aumento dei livelli circolanti del glicopeptide, in caso di riduzione del filtrato glomerulare di grado severo, secondario a ridotta clearance renale
