# Supplementary material for: Attuali applicazioni della determinazione dei livelli plasmatici di copeptina in contesti non-endocrinologici
Source: L'Endocrinologo. 2022 Nov 8;23(6):592–7. [Article in Italian] doi: 10.1007/s40619-022-01180-8 (PMC9641695; doi:10.1007/s40619-022-01180-8)
Supplement: Supplementary file 3 [file 40619_2022_1180_MOESM3_ESM.doc]

| **QUADRO SINOTTICO DEI FARMACI CITATI** | | | | |
| --- | --- | --- | --- | --- |
| **Principio attivo** | **Nome commerciale** | **Ditta produttrice** | **Formulazione** | **Dosaggi disponibili in commercio** |
| Tolvaptan | Jinarc® | Otsuka Pharmaceutical | Compresse | *Dose frazionata* 45 mg + 15 mg  60 mg + 30 mg  90 mg + 30 mg  *Dose ridotta*  30 mg  15 mg |

Per l’elenco completo dei farmaci citati e le relative schede tecniche si rimanda al prontuario dei farmaci
